# A novel scrolling text reading paradigm for improving the performance of multiclass and hybrid brain computer interface systems

**DOI:** 10.1371/journal.pone.0334784

**Published:** 2025-11-13

**Authors:** Ebru Ergün, Önder Aydemir, Onur Erdem Korkmaz

**Affiliations:** 1 Department of Electrical and Electronics Engineering, Recep Tayyip Erdogan University, Rize, Turkey; 2 Department of Electrical and Electronics Engineering, Karadeniz Technical University, Trabzon, Turkey; 3 Karadeniz Technical University, Medical Device Design and Production Application and Research Center, Trabzon, Turkey; 4 Department of Electrical and Electronics Engineering, Atatürk University, Erzurum, Turkey; Columbia University, UNITED STATES OF AMERICA

## Abstract

A Brain-Computer Interface (BCI) enables direct communication between the brain and external devices, such as computers or prosthetic limbs. This allows the brain to send commands while receiving sensory feedback from the device. Despite their potential, the performance limitations of existing BCI systems have motivated researchers to improve their efficiency and reliability. To address this challenge, the present study introduces a novel BCI paradigm centered on a cognitive task involving the reading of scrolling text in four different directions: right, left, up and down. The primary objective was to explore the electroencephalography (EEG) and near-infrared spectroscopy (NIRS) signals within this framework and assess the potential of hybrid BCI systems based on this innovative paradigm. The experimental protocol involved eight participants performing tasks across four classes of scrolling text. To optimize system accuracy and speed, EEG and NIRS data were segmented into discrete temporal windows. Features were extracted using the Hilbert Transform, while classification was performed via the k-nearest neighbor algorithm. The proposed approach achieved a classification accuracy of 96.28% ± 1.30% for multi-class tasks, demonstrating the effectiveness of hybrid modalities. This study not only introduces a novel paradigm for hybrid BCI systems, but also validates its performance, providing a promising direction for advancing the field.

## Introduction

The primary characteristic of human society is its capacity for communication and social interaction, which enables individuals to share emotions, desires and innovative ideas with others [1,2]. Communication becomes more natural and less constrained when it is facilitated by speech, gesture or writing. However, people with amyotrophic lateral sclerosis (ALS), who experience severe movement impairments despite no direct motor neuron dysfunction, often lack access to these conventional means of interaction. For such individuals, communication with others is almost impossible, severely limiting their engagement with the environment. Brain-computer interface (BCI) systems address this challenge by providing a direct communication link between the brain and external devices. BCI systems were initially developed to assist people with ALS and similar conditions, enabling them to overcome limitations in muscle control. These systems empower users by facilitating interaction with computers, operation of assistive technologies such as robotic arms or wheelchairs, and engagement in daily activities, thereby increasing autonomy and quality of life [3–5]. Over time, as scientific research has progressed, BCI applications have expanded beyond medical use into areas such as entertainment, the arts and other non-medical fields. BCIs use signals recorded by various neuroimaging techniques, including electroencephalography (EEG), near-infrared spectroscopy (NIRS), electrocorticography (ECoG), magnetoencephalography, functional magnetic resonance imaging and positron emission tomography [6]. Each method has its own advantages and limitations. For example, while ECoG has an excellent signal-to-noise ratio and is suitable for long-term use, it is highly invasive and carries significant risks due to the implantation of microelectrodes. Conversely, EEG is non-invasive, cost-effective, and provides high spatial resolution, but is highly susceptible to external and internal noise sources such as electrical interference and body motion [7,8]. NIRS, on the other hand, is less affected by body motion and electrical interference, but suffers from limited penetration depth. Given these trade-offs, combining multiple signal acquisition methods to mitigate their individual drawbacks may improve BCI performance. Despite its limitations, EEG remains the preferred choice in BCI studies due to its many advantages. However, evidence from the literature suggests that EEG-based BCI systems alone often fail to achieve satisfactory performance, highlighting the need for improvements in both speed and accuracy. This has led researchers to focus on optimizing the experimental paradigms and mathematical models used in BCI studies [9]. The present study aimed to improve the performance of a hybrid EEG+NIRS-based BCI system by introducing an original experimental paradigm while adhering to established mathematical models in the field.

In recent years, there has been growing interest in BCI systems using motor imagery (MI), P300 and steady-state visual evoked potential (SSVEP) approaches [10,11]. While MI-based systems have the notable advantage of relying solely on EEG as an input signal, they have yet to demonstrate consistently high performance. Similarly, P300 and SSVEP-based systems, which also rely solely on EEG and require minimal user training, have shown only partial efficacy in existing studies [12]. Among these studies, Yu et al. introduced a hybrid BCI system that integrates MI and P300 event-related potential to enable the asynchronous operation of a robotic wheelchair. This system allows users to perform self-paced control tasks by alternating between MI tasks and focusing on P300 visual stimuli, providing eleven distinct commands for movements, speed adjustments, and stops. Their approach highlights the feasibility of sequential mental task processing for practical applications in assistive technologies [13]. Despite some promising results, these systems face a critical limitation: the user’s brain response to stimuli tends to diminish over time. As users become accustomed to infrequent stimuli and develop familiarity with the system, the performance of these systems gradually declines [14]. Given these challenges, there is a clear gap in the literature in terms of both experimental paradigms and the variety of signal types used in BCI research. To address this limitation, the current study introduces a novel experimental paradigm. This paradigm was implemented using a hybrid EEG+NIRS dataset, providing a multidimensional approach to BCI system design. Supported by Karadeniz Technical University Scientific Research Projects Coordination Unit under project number FHD-2020–9166, a four-class scrolling text reading dataset was recorded at Atatürk University. The features extracted from this hybrid dataset, which includes the original experimental paradigm, were then classified to develop a high-performance BCI system. This approach not only broadens the range of experimental paradigms in BCI research, but also increases the robustness of the system by integrating hybrid modalities.

Building on advances in hybrid BCI systems, considerable effort has been directed towards exploiting the combined strengths of EEG and NIRS signals to improve classification accuracy (CA). Over the past decade, researchers have explored various approaches, including the classification of mental arithmetic (MA), motor execution (ME), and MI-based 2-class or multi-class signals. For example, Al-Quraishi et al. demonstrated the effectiveness of a hybrid system using a 2-class ME dataset recorded from 20 subjects. Their approach involved extracting statistical features such as variance, kurtosis and skewness from NIRS signals, and features such as mean absolute value and root mean square from EEG signals. Using a support vector machine (SVM) classifier, they achieved a CA of 93.01% [15]. Similarly, Li et al. conducted an online study on 2-class EEG+NIRS signals, extracting wavelet transform features from EEG and mean-based features from NIRS, and achieved a CA of 91.02% using SVM [16]. Furthermore, Al-Shargie et al. focused on a 2-class MA-based dataset, using mean concentration features for HbO from NIRS and mean power from EEG. Their approach yielded a CA of 95.10% with SVM classification [17]. Meanwhile, Cicalese et al. extended the scope to a 4-class MA-based dataset, using averaged NIRS features and band power-based EEG features. Using linear discriminant analysis (LDA), they achieved a CA of 79.31% [18]. In another study, Blokland et al. investigated a hybrid EEG+NIRS system for brain-computer interfaces in tetraplegia. They achieved 79.00% accuracy for attempted and 70.00% for imagined movements in patients, while controls reached 87.00% for actual and 79.00% for imagined movements [19]. Together, these studies highlight the potential of hybrid BCI systems, although the performance metrics suggest for further refinement.

Evaluating the current state of hybrid BCI systems, it is clear that achieving higher CA depends heavily on extracting effective features that are intrinsically linked to the brain’s neural activity during different mental tasks. Given that specific brain regions are activated by tasks involving visual, auditory and arithmetic functions, it is plausible to design paradigms that effectively engage these areas. Motivated by these insights, the present study introduces a groundbreaking experimental paradigm designed to activate distinct brain regions by engaging participants in the cognitively demanding task of reading scrolling text in four directions: right, left, up and down. This paradigm is notable for its ability to systematically stimulate specific neural areas associated with visual processing and cognitive control, allowing a more comprehensive exploration of hybrid EEG+NIRS signals. By exploiting this unique task, the study ensures robust and distinctive neural activations, providing a significant advantage in feature extraction and classification. The paradigm’s innovative approach to eliciting directional cognitive responses is a key differentiator, as it not only improves the signal-to-noise ratio, but also enables the generation of features with enhanced discriminative power. Hilbert Transform (HT)-based features were carefully extracted from the resulting EEG+NIRS signals, capturing nuanced temporal and frequency domain characteristics. Classification, performed using the k-nearest neighbor (k-NN) algorithm, demonstrated the effectiveness of the paradigm in translating these complex features into a high-performance hybrid BCI system. Furthermore, the proposed paradigm introduced a level of methodological rigor by incorporating hybrid modalities, such as EEG combined with oxygenated (Oxy) and deoxygenated (Deoxy) hemoglobin signals, while also evaluating each modality independently. This dual capability highlights the versatility of the paradigm and its potential to push the boundaries of hybrid BCI systems, providing an invaluable framework for improving CA and system reliability. The analysis was performed using MATLAB 2020, and the results were compared to commonly used features and classifiers in the literature. This comprehensive evaluation confirmed the superiority of the proposed paradigm, which achieved a CA of 96.28%. These results highlighted the significant contribution of the proposed four-class scrolling text paradigm to the performance and reliability of EEG+NIRS-based BCI systems, setting a new benchmark in hybrid BCI research.

## Materials and methods

### Data acquisition and experimental procedure

**Fig 1:**
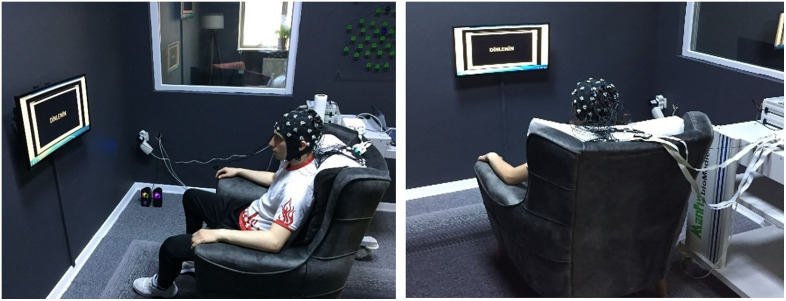
Illustration of a subject participating in the experimental procedure.

**Fig 2:**
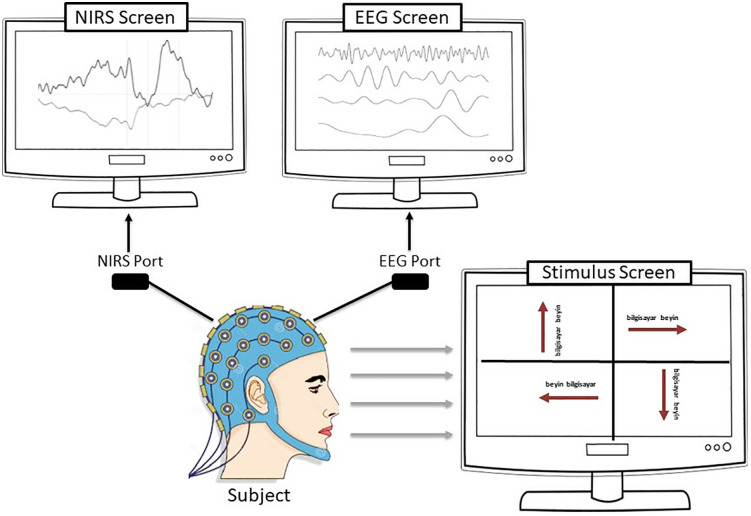
The experimental setup used for simultaneous EEG and NIRS signal acquisition.

**Fig 3:**
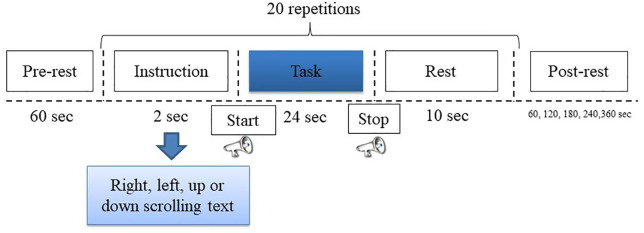
A schematic diagram of the experimental paradigm illustrating the sequence and structure of the experiment.

The EEG+NIRS dataset was collected at Atatürk University, Faculty of Sports Science. Eight healthy volunteers (five males and three females), aged between 19 and 38 years, participated in the experiments conducted from January 3, 2022, to April 15, 2022. Prior to the commencement of the study, all participants were thoroughly informed about the experimental procedures, potential risks, and their rights, both verbally and through detailed written information forms. Subsequently, written informed consent was obtained from each participant to ensure voluntary participation in accordance with ethical standards. Additionally, verbal confirmation of consent was secured immediately before the experimental sessions to reaffirm willingness to participate. The study protocol was reviewed and approved by the Karadeniz Technical University Ethics Committee, guaranteeing adherence to ethical guidelines and protection of participants’ rights throughout the research process. The study was conducted according to the tenets of the Declaration of Helsinki [20] and was approved by the Ethics Committee of Karadeniz Technical University. For this study, the subjects were designated K1, K2, K3, K4, K5, K6, K7 and K8, respectively. The experimental paradigm chosen for this study was: “reading text scrolling to the right, left, up or down”. The aim of the experiment was to learn how the reading of text in different directions produces different signals in the brain. The brain processes visual information and produces motor responses in a complex way. This involves the visual cortex, neural circuits that control eye movements, motor regions and attention systems. These mechanisms work together to interpret text scrolled in different directions by stimulating different motor regions depending on the direction of scrolling. In order to be able to focus on the text and to improve comprehension, attentional processes are essential. The complex interaction of several brain networks and circuits is involved in the processing of visual information, the control of eye movements and the generation of motor responses. Before the experiment, the experimental methods and steps were explained to each participant and it was ensured that all participants fully understood the whole process. The person conducting the experiment was responsible for monitoring the experimental process to ensure reliability. The experiment was conducted in a large, enclosed laboratory. Subjects were seated comfortably in a chair approximately one meter away from a 1920×1080 resolution LCD monitor, as shown in Fig 1. The subjects were provided with sufficient breaks to maintain their physical and mental condition and to ensure a high quality of the signal. The data were collected by means of the experimental set-up shown in Fig 2. The figure shows that brain signals were recorded synchronously using EEG and NIRS electrodes attached to the skull. This was done while the subject was looking at the stimulus screen. The EEG and NIRS ports were used to transmit the information from the EEG and NIRS electrodes separately to a computer screen. After providing a suitable experimental environment, EEG+NIRS signals based on reading moving text to the right (class 1), left (class 2), up (class 3) and down (class 4) were recorded from the subjects. The experimental procedure for a single session of the experiment was as shown in Fig 3.

The experimental protocol consisted of six sessions for each participant, with each session comprising 20 trials. Each session commenced with a 60-second resting period to establish a physiological baseline. This was followed by a 2-second task cue phase, during which a visual instruction appeared at the center of the screen, indicating the direction in which the upcoming scrolling text would move. Task directions were presented in a randomized manner, ensuring an equal distribution across all directions. After the task cue, a short auditory beep marked the beginning of the 24-second task execution phase. During this phase, participants were asked to read the scrolling text as it moved in the instructed direction. At the end of each task phase, another beep indicated the start of a 10-second inter-trial resting period before the next trial began. To mitigate fatigue and maintain attention throughout the experiment, scheduled breaks were incorporated between sessions. As shown in Fig 3, the inter-session intervals increased progressively across sessions, ranging from 60 seconds in the first session to 360 seconds in the final one. These gradually lengthened breaks were designed to allow both physical and cognitive recovery, thereby minimizing attentional drift and preserving signal quality. The overall session structure was intentionally designed to gradually acclimate participants to the task demands while preventing cognitive overload.

The total experimental duration for each subject was 12480-sec. To manage potential fatigue and maintain concentration, sufficient breaks were provided between sessions. These breaks allowed participants to recover both physically and mentally, minimized attentional lapses and ensured consistent signal quality throughout the experiment. The structured design of the sessions, with increasing task duration, aimed to gradually acclimate participants to the task demands without overburdening them. The engaging nature of the scrolling text task, coupled with visual cues and auditory signals, further enhanced participant concentration and reduced the likelihood of attention loss. By integrating these measures, the study ensured that the extended duration of the experiment did not compromise its outcomes. The high-quality signals and successful completion of the experiment by all participants underscore the effectiveness of this carefully calibrated experimental design.

**Fig 4:**
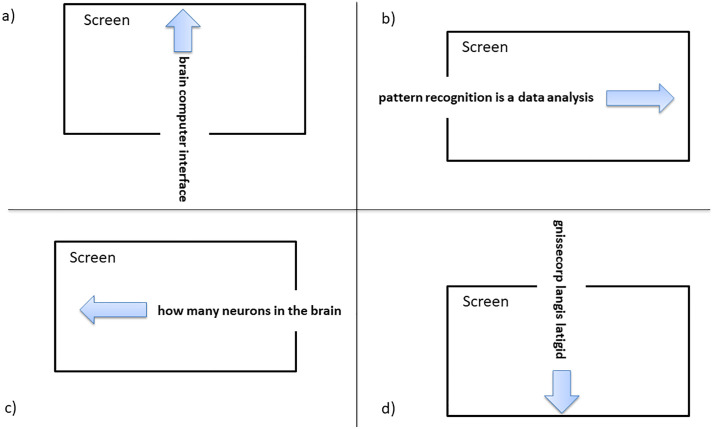
The visual screen displaying the scrolling text used during the experiment. The text flows in one of four directions-right, left, up or down-depending on the task instructions given at the beginning of each trial.

In Fig 3, the subject was given information about the direction in which the scrolling text was to be read. During the task, they attempted to follow the scrolling text displayed on a screen similar to the one shown in Fig 4. During the experiment, the scrolling text contained different scientific sentences for each trial. The scrolling text moved at the same speed for each trial. During the experiment, participants read scientific sentences from the screen. These sentences were randomly selected from different topics of the Science and Technology Magazine of the Technological Research Council of Turkey (TUBITAK) [21].

**Fig 5:**
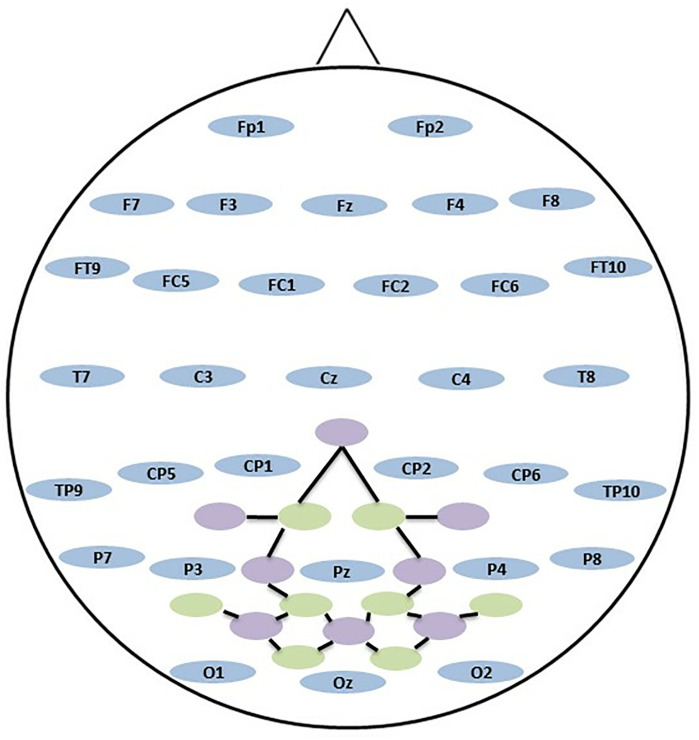
Illustration of EEG electrode positions next to NIRS emitters and detectors. The EEG electrodes, NIRS detectors and NIRS emitters are represented by blue, purple and green circles, respectively. NIRS channels are represented by black solid lines.

While the EEG signals were recorded using the ActiChamp device developed by Brain Product, the NIRS signals were recorded at the same time using the NIRX optical neuroimaging device. A single hat was used to place the EEG and NIRS electrodes on the subjects’ skulls. Electrodes were placed according to the international 10/20 system and unipolar EEG recording was performed during the experiments. During data collection, all electrode impedances were kept below 5 kΩ. EEG electrodes were placed according to the international 10/20 system with 31 electrodes in the frontal, central, temporal and occipital regions. In addition, 18 channels, consisting of 8 emitters and 8 detectors, were used to acquire NIRS data. The central and occipital regions where vision is active are covered by NIRS emitters and detectors. The general view of the electrodes, detectors and emitters is the same as that shown in Fig 5. The figure shows the spatial arrangement of the EEG electrodes, to ensure optimal coverage of brain regions associated with the experimental tasks. In addition, the positions of the NIRS emitters and detectors are shown, strategically aligned to record hemodynamic responses from corresponding cortical areas.

Subjects were explicitly instructed to maintain a stable posture throughout the experiment. They were seated comfortably in a fixed position to ensure that neither head nor trunk rotation was allowed to improve the readability of the vertically scrolling text. This controlled setup was implemented to minimize any extraneous physical movements that could potentially affect the brain signals being recorded. By maintaining this restriction, the experiment ensured that the EEG+NIRS signals primarily reflected cognitive and neural activity associated with the visual task itself, rather than being confounded by physical adaptations or compensatory behaviors. In addition, the scrolling text was designed at a font size and speed optimized for clear visibility without the need for physical repositioning, ensuring consistency of reading performance across participants.

## Methods

**Fig 6:**
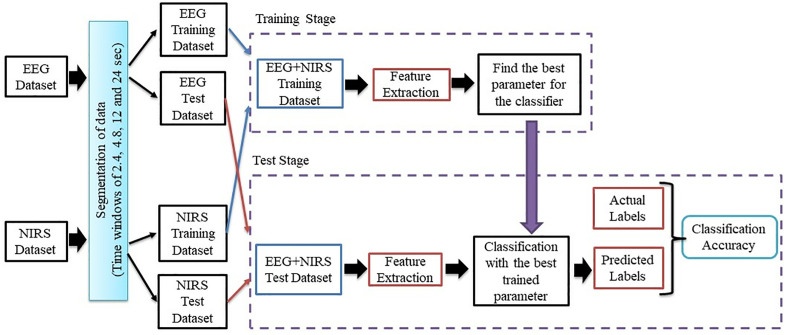
General flow chart of the proposed method.

The proposed method involves a systematic approach that includes several key stages: data acquisition, segmentation of data time windows, feature extraction and classification. These stages are interrelated, as shown in the general flowchart in Fig 6. Initially, EEG and NIRS signals were recorded from subjects during task performance for four different classes. Once recorded, the signals were pre-processed to ensure the removal of unwanted noise, in particular power line interference, using a notch filter. The EEG and NIRS signals were then segmented into time windows to enhance the data for subsequent analysis. The time windows were carefully chosen to be multiples of the task duration, specifically 2.4, 4.8, 12 and 24-sec, to match the 24-sec task intervals. The segmented data sets containing both EEG and NIRS signals were then randomly divided into training and test sets, with 75% allocated to training and 25% reserved for testing. It is important to note that both training and test sets contained an equal number of trials for each class to maintain consistency and balance in the dataset. To enhance methodological transparency and control for subject-specific biases, a leave-one-subject-out cross-validation strategy was implemented. Feature extraction followed, where features were derived from the segmented signals using selected mathematical methods. In this study, features based on HT, Fast Walsh-Hadamard Transform (FWHT), Band Power (BP) and various Statistical Features (SF) were extracted. These extracted features were then fed into a series of machine learning algorithms for classification. In order to achieve more robust and reliable results, classification was performed using four different algorithms: k-NN, SVM, Decision Tree Algorithm (DTA), Random Forest Algorithm (RFA) and Short-Term Memory (LSTM). The performance of each classifier was evaluated by averaging the CA results, and the entire training and testing procedure was repeated 50 times to ensure the reliability and consistency of the results. The EEG data consisted of 120 trials recorded from 30 channels at a sampling rate of 250 Hz, with each trial lasting 24-sec. In parallel, the NIRS data set consisted of 120 trials and 18 channels at a sampling rate of 10.17 Hz, also with a duration of 24-sec, resulting in a data size of 120×samples(EEG or NIRS)×channels. All computational processes, including data segmentation, feature extraction and classification, were performed on a system equipped with 16 GB of RAM and a 2.92 GHz Intel Core i7 processor, ensuring efficient handling of the dataset and the algorithms.

### Feature extraction

Feature extraction refers to the process of extracting distinct features from a data set using statistical or mathematical methods with the aim of distinguishing between classes. In this study, we first addressed the removal of unwanted noise from the EEG and NIRS signals by implementing a 2nd order band-stop filter for both modalities. For the EEG signals, a band-stop filter was designed to target the power line noise typically occurring at 50 Hz, with a frequency range between 48 Hz and 53 Hz. This filter was configured using the Butterworth design method, known for its smooth frequency response and minimal signal distortion, and the sampling rate was adjusted to match the EEG recording conditions. Similarly, a band-stop filter was applied to the NIRS data in the frequency range 0.1 Hz to 5 Hz, effectively filtering out low-frequency physiological noise such as motion artefacts and heart rate fluctuations. The same Butterworth design method was used to ensure optimum performance while maintaining signal integrity. Both filters were carefully tailored to the specific noise characteristics of each dataset, facilitating the extraction of cleaner data for subsequent analysis and classification. In addition, the modified Beer-Lambert law (MBL) was applied to the NIRS signals in this study to measure changes in hemoglobin concentration. The MBL is derived from the extinction coefficient of the absorbing species and the path length of the scattered light as described by the following equation [22]. Hemoglobin exchange for Oxy and Deoxy hemoglobin was calculated using this law, providing essential information for further feature extraction and analysis. The general expression of this law is given by


$$\left[\begin{array}{c} {\Delta c}_{O}(z)\\ {\Delta c}_{D}(z) \end{array}]=\frac{\text{1}}{v\times du}\;[\begin{array}{cc} \beta_{O}\left(\lambda_{\text{1}}\right) & \beta_{D}\left(\lambda_{\text{1}}\right)\\ \beta_{O}\left(\lambda_{\text{2}}\right) & \beta_{D}\left(\lambda_{\text{2}}\right) \end{array}]-\text{1}[\begin{array}{c} \Delta B\left(z,\lambda_{\text{1}}\right)\\ \Delta B\left(z,\lambda_{\text{2}}\right) \end{array}\right]$$ (1)

Here, for i= 1,2, $\Delta B(z,\;\lambda_{i})$; represents the optical intensity variation of an emitter at a wavelength $\lambda_{i}$. $\beta_{O/D}(\lambda_{i})$ is the extinction coefficient of Oxy and Deoxy du is the differential path length factor between the emitter and detector, and v represents the distance between them [23]. After applying the MBL to the NIRS data, features were extracted from the HT, FWHT, BP and SF. HT originated in the work of Hilbert in 1905 on a problem concerning analysis functions called the Riemann – Hilbert problem [24]. HT is a method for obtaining the minimum phase response from spectral analysis. The types of signal that make up brain dynamics, such as the EEG or NIRS, are non-stationary. However, HT is a nonlinear operator. It allows non-stationary signals to be identified and studied by representing frequency as a rate of change of phase over time [25]. Typically, raw data contains many frequencies changing over time. HT has a particularly simple representation in the frequency domain: it introduces a phase shift of ±90∘, depending on the sign of each of the frequency components of a function. The HT of the signal y(t), representing the EEG or NIRS signal in this study, is obtained by convolving the signal with $h(t)=\frac{\text{1}}{\pi t}$ and it is given by


$$H\left\{y(t)\right\}=\hat{yn}(t)=\text{y}(t)*\frac{\text{1}}{\pi t}=\frac{\text{1}}{\pi }\int_{-\infty }^{\infty }\frac{\text{y}(\tau )}{t-\tau }d\tau$$ (2)

The impulse response of the h(t) is defined as


H(ω)=F[h(t)]=−i·sign(ω) (3)

where the impulse response of the signal is +i for ω<0 and −i for ω>0. Therefore, HT has a shifting effect of +90 degrees for negative frequency components of y(t) and −90 degrees for positive frequency components. Due to causality, the HT of a signal y(t) is associated with its real and imaginary parts, can be quantified as


$$\hat{yn}(t)=r(\hat{yn}(t))+i(\hat{yn}(t))$$ (4)

The average of the square root of the sum of squares of the real and imaginary parts of $\hat{yn}(t)$, denoted as (${HT}_{abs}$), is given in


$${HT}_{abs}=\;\frac{\sum_{i=\text{1}}^{n}\sqrt{{r({\hat{yn}}_{i}(t))}^{\text{2}}+{i({\hat{yn}}_{i}(t))}^{\text{2}}}}{n}$$ (5)

where n represents the length of $\hat{yn}(t)$. In this study, the (${HT}_{abs}$) values have been used as features representing EEG, Deoxy, and Oxy trials. One of the main reasons for the effective use of HT as a feature extraction method in EEG and NIRS is the analysis of the frequency domain. The EEG typically contains brain waves that manifest themselves in specific frequency ranges, such as alpha waves, beta waves and theta waves. HT can do a detailed examination of the frequency characteristics of brain activity through the extraction of these frequency components. NIRS measures changes in tissue absorption for both oxygenated and deoxygenated blood. HT can be used to analyze these data in the frequency domain, providing insight into the dynamic changes in blood oxygenation over time [26]. In addition, HT can extract phase changes in EEG and NIRS signals. NIRS provides valuable insight into instantaneous oxygenation and coordination between tissues, while phase changes in EEG provide important information about cognitive processes. Furthermore, HT generates the analytic representation of the original signals, providing a more suitable form for analyzing by representing the signals as complex numbers [27].

### Classification

Classification is simply the procedure of dividing data into different predefined categories within a data set. Classification algorithms learn this distribution model from a specific training set of data and then attempt to correctly classify unknown test data when it is presented to them. The values that indicate these categories in the data set are called labels, and they are used to determine the category of the data during the training and the test [28]. A confusion matrix in machine learning visualizes classification performance and allows comparison of different algorithms. In multi-class problems, the matrix has dimensions of N×N, where N is the number of class labels, and metrics are calculated for each class based on specific features. These metrics can then be combined to compute the overall CA for the entire matrix [29]. The equality for CA in a multi-class problem, calculated using the confusion matrix in Table 1, is provided in

**Table 1:** Confusion matrix for multiclass classification.

Confusion Matrix		Predicted Classes			
		${\mathrm{\backslash bold}Z}_{\text{1}}$	${\mathrm{\backslash bold}Z}_{\text{2}}$	…	${\mathrm{\backslash bold}Z}_{\mathrm{\backslash bold}N}$
**Actual Classes**	${\text{Z}}_{\text{1}}$	${\text{Z}}_{\text{1},\text{1}}$	FP	…	${\text{Z}}_{\text{1},N}$
	${\text{Z}}_{\text{2}}$	FN	TP	…	FN
	…	…	…	…	…
	${\text{Z}}_{N}$	${\text{Z}}_{N,\text{1}}$	FP	…	${\text{Z}}_{N,N}$


$$CA\;=\;\frac{\sum_{i=\text{1}}^{N}TP({\text{Z}}_{i})}{\sum_{i=\text{1}}^{N}\sum_{j=\text{1}}^{N}.{\text{Z}}_{i,j}}x\text{100}$$ (6)

The k-NN is a supervised machine learning algorithm. It performs the classification process by determining the label of a test trial based on its nearest neighbor in the train set. The objective of the method is to find the distance/similarity between the train and test trials. In this study, the Euclidean distance, the cosine distance, the city block distance and the correlation of these distance measures were used [30,31]. The Euclidean distance gave the best results among them. The Euclidean distance (ED) between two studies is calculated by


$$ED(g,h)=\sqrt{\sum_{i=\text{1}}^{n}{\left(p_{gi}-p_{hi}\right)}^{\text{2}}}$$ (7)

where the Euclidean distance between n points g and h is expressed as ED. One of the important aspects of the k-NN method is to determine the value of the k parameter. In this study, the optimal value of k for the k-NN algorithm was determined using a systematic random subsampling approach. For each subject and modality, the dataset was repeatedly divided into training and validation subsets to evaluate different k values. This method ensured the selection of an optimal k that minimized classification errors while maintaining generalization to unseen data. The robustness of this approach was verified by consistent classifier performance over multiple runs. To account for the inherent variability in EEG and NIRS data, the optimal k was recalculated for each modality, taking into account subject-specific differences. The non-parametric nature of k-NN allows it to deal effectively with noisy data by averaging over a wider neighborhood for larger k values, thereby mitigating the effect of outliers. In addition, pre-processing steps were used to standardize the data, thereby increasing the stability of the classification in the presence of noise. Ultimately, this iterative validation process ensures reliable performance on diverse and noisy datasets.

### Representation of experimental results

**Fig 7:**
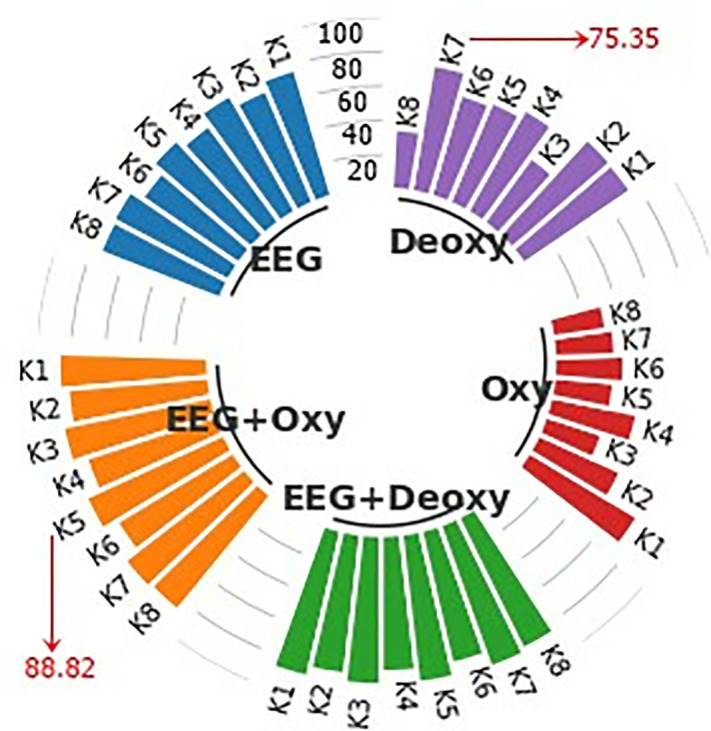
Display of subject-specific bar graphs showing average CA as percentages across different modalities.

An example of a graph used for presenting the results in this study is the subject-specific bar graphs shown in Fig 7. Each graph contains the average CA results of 50 runs for 8 subjects in the EEG+Deoxy, EEG + Oxy, EEG, Deoxy, and Oxy modalities.

Each column in the bar graph represents the CA for each subject, specified as $K_{\text{1}},\;K_{\text{2}},\;K_{\text{3}},K_{\text{4}},K_{\text{5}},K_{\text{6}},K_{\text{7}}$ and $K_{\text{8}}$, representing Subject 1, Subject 2, Subject 3, Subject 4, Subject 5, Subject 6, Subject 7, and Subject 8, respectively. The scale lines between the bar columns represent boundary lines with values of 20, 40, 60, 80, and 100 indicating the CA. As shown in this example graph, an average CA of 88.82% was obtained for Subject 5 in the EEG + Oxy modality, while a similar calculation resulted in a CA of 75.35% for Subject 7 in the Deoxy modality. Furthermore, considering the experiments were conducted with 8 different subjects, all the experimental results, including subject-based train and test CA results for each modality, were presented using these graphs to enhance the visual representation of the findings.

## Results

This study introduced an innovative hybrid BCI system combining EEG and NIRS modalities based on a scrolling text reading paradigm. To evaluate the system, a comprehensive dataset was generated by recording brain signals from participants reading scrolling text in four directions, as shown in Fig 3. The data set was segmented into four different time periods: 2.4, 4.8, 12, and 24-sec. Each segment underwent feature extraction using HT, and the k-NN algorithm was used for classification. The dataset was divided into training (75%) and test (25%) subsets, with analyses performed for individual and combined EEG and NIRS modalities across all subjects, averaging results over 50 runs.

### Classification performance for 2.4-second segments

**Fig 8:**
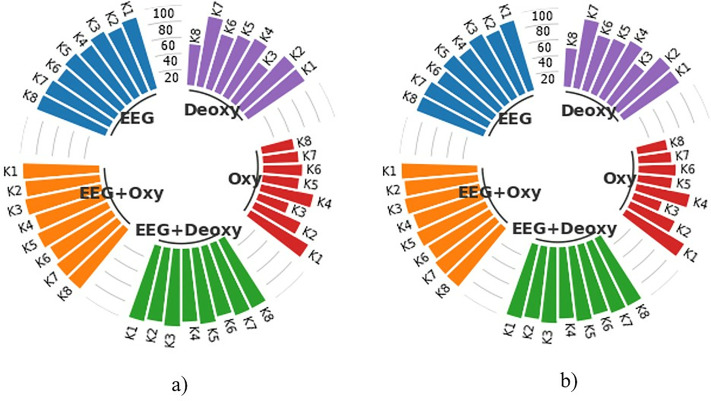
Average CA Results for 2.4-Second Time Segments: a) Training Set and b) Test Set, representing both single and hybrid modalities in terms of percentage.

The average training and test CA results for each modality are shown in the subject-based bar chart in Fig 8. These results were obtained by classifying HT-based features extracted from 2.4 s time segments with k-NN for each subject in 50 runs. As shown in Fig 8, the best test CAs for the EEG+Deoxy and EEG + Oxy modalities were calculated to be 98.45% ± 0.94 and 98.47% ± 1.33 for K3, respectively. The worst test CAs for these modalities were 92.74% ± 1.70 for K4 and 92.95% ± 1.49 for K6. On the other hand, the best test CAs for the EEG, Deoxy and Oxy modalities were 94.26% ± 2.14 for K3, 90.30% ± 2.17 for K7 and 87.58% ± 1.90 for K1, respectively. The worst test CAs for these modalities were 86.55% ± 1.49 for K6, 51.32% ± 3.46 for K8 and 39.33% ± 2.37 for K3.

### Classification performance for 4.8-second segments

**Fig 9:**
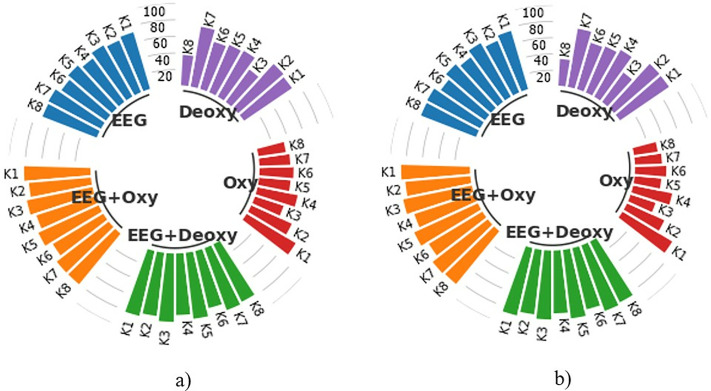
Average CA Results for 4.8-Second Time Segments: a) Training Set and b) Test Set, representing both single and hybrid modalities in terms of percentage.

The subject-based bar chart in Fig 9 provides a detailed comparison of the average train and test CAs for each modality following the classification of HT-based features extracted from 4.8-sec time segments using k-NN over 50 runs for each subject. As can be seen in Fig 9, the best test CAs for the EEG+Deoxy and EEG + Oxy modalities were calculated to be 88.41% ± 3.56 for K7 and 88.83% ± 3.88 for K3, respectively. The worst test CAs were 80.01% ± 4.07 for K6 and 79.61% ± 3.87 for K6. In addition, the best test CAs were 78.47% ± 3.94 for K3, 76.46% ± 3.91 for K2 and 74.06% ± 3.77 for K1 for the EEG, Deoxy and Oxy modalities, respectively. The worst test SDs were 68.62% ± 3.87 for K6, 35.63% ± 4.48 for K8 and 32.16% ± 4.27 for K8, respectively.

### Classification performance for 12 and 24-second segments

**Fig 10:**
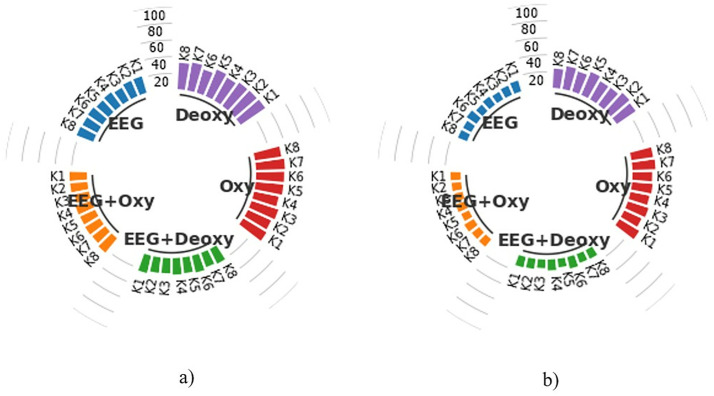
Average CA Results for 12-Second Time Segments: a) Training Set and b) Test Set, representing both single and hybrid modalities in terms of percentage.

**Fig 11:**
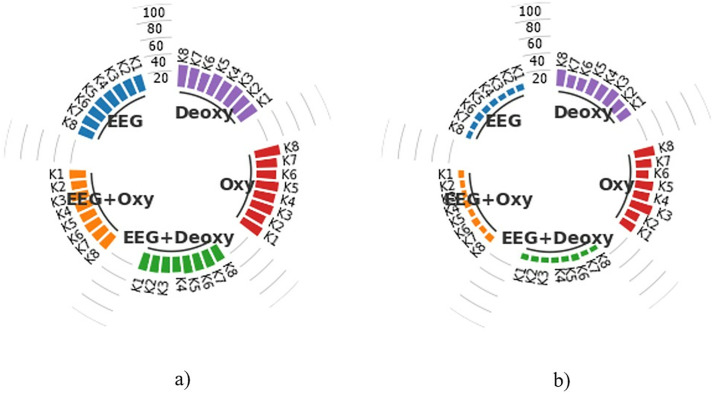
Average CA Results for 24-Second Time Segments: a) Training Set and b) Test Set, representing both single and hybrid modalities in terms of percentage.

The average train and test CA results for each subject were obtained by classifying HT-based features extracted from 12-sec and 24-sec time segments with k-NN in 50 runs. These results are presented in Fig 10 and Fig 11. As seen in Fig 10, the test CA for the EEG+Deoxy and EEG + Oxy modalities ranges from 16.85% ± 3.70 to 13.02% ± 3.06 and 15.90% ± 3.69 to 12.80% ± 2.82 between subjects, respectively. On the other hand, the EEG, Deoxy, and Oxy modalities, the test CAs range from 16.85% ± 4.37 to 13.00% ± 2.82, 32.20% ± 4.60 to 25.55% ± 4.24 and 30.02% ± 4.54 to 25.40% ± 4.63 between subjects, respectively. Looking at Fig 11, the test CA for the EEG+Deoxy and EEG + Oxy modalities ranges from 10.75% ± 4.95 to 8.70% ± 3.75 and 11.10% ± 3.85 to 8.55% ± 4.63 between subjects, respectively. On the other hand, the EEG, Deoxy, and Oxy modalities, the test CAs range from 11.15% ± 4.74 to 8.90% ± 3.85, 24.00% ± 6.26 to 17.85% ± 5.86, and 29.45% ± 7.11 to 17.75% ± 6.08 between subjects, respectively.

### Evaluation of results based on time segments

When analyzing the results for all time segments, it can be seen that the most effective results are obtained with 2.4-sec time segments. The average train and test CAs are calculated for 8 subjects using the HT features extracted from the 2.4-sec time segments and classified with k-NN. The train CA is 96.01% ± 2.26 and the test CA is 96.23% ± 1.30 for the EEG+Deoxy modality. The train CA is 95.89% ± 2.13 and the test CA is 96.28% ± 1.30 for the EEG + Oxy modality. For the EEG modality the train CA is 91.63% ± 6.16 and the test CA is 91.25% ± 2.64. For the Deoxy modality the train CA is 77.18% ± 4.44 and the test CA is 77.31% ± 2.53. For the Oxy modality the train CA is 58.66% ± 4.39 and the test CA is 57.67% ± 2.75. It is important to note that with a test CA of 96.28% ± 1.30, the most effective results are obtained with the EEG + Oxy modality. Furthermore, if we compare the hybrid modality with the single modalities, we can see that the results obtained with the hybrid modality are 5.03% higher than those obtained with EEG, 18.97% higher than those obtained with Deoxy and 41.07% higher than those obtained with Oxy. Examining the results in Fig 8, it can be seen that test CA for subjects K1, K3, K5, K7 and K8 is better for EEG+Deoxy, EEG + Oxy and EEG modalities than other subjects. However, for the same modalities, the test CA is relatively lower for subject K6. This could be due to the fact that he was not able to concentrate fully during the experiment. This variability in performance, particularly for subject K6, may be attributed to factors such as fluctuations in attention, fatigue, or individual differences in neural responsiveness during the task.

Furthermore, by employing the HT on 2.4-second data segments and using the k-NN classifier, we systematically evaluated the classification performance of different combinations of EEG and NIRS. Our results revealed that the highest accuracy, reaching 96.28%, was achieved through the integration of EEG and Oxy signals. This finding suggests that oxygenated hemoglobin provides complementary and supportive information to the EEG signal by sensitively reflecting cerebral blood flow changes during cognitive tasks. In contrast, while the EEG signal alone demonstrated a respectable accuracy of 91.25%, the hemoglobin signals by themselves yielded considerably lower performance, with Oxy alone achieving only 55.21%. This indicates that hemoglobin signals in isolation may lack sufficient discriminatory power but, when combined with EEG, offer richer and more meaningful neurophysiological insights. Additionally, the Deoxy signal exhibited moderate performance on its own, implying that it captures some task-related neural variations, although not enough to achieve high classification accuracy independently. In summary, the high temporal resolution of EEG signals combined with the metabolic information carried by hemoglobin signals clearly demonstrates that their integration enables more accurate classification of diverse cognitive tasks. This underscores the practical superiority of hybrid systems in brain-computer interface applications.

Furthermore, the F1-score, recall, and precision metrics were additionally calculated based on HT features classified with the k-NN algorithm for each of the eight participants to rigorously assess the multi-class classification performance. The F1-scores ranged from 93.08% to 98.07%, recall values spanned 93.07% to 98.11%, and precision values varied between 93.81% and 98.15%. Participant K1 exhibited the highest performance with an F1-score of 98.07%, recall of 98.11%, and precision of 97.89%. In contrast, participant K6 showed the lowest metrics with an F1-score of 93.23%, recall of 93.07%, and precision of 93.81%, reflecting inter-subject variability likely related to attentional fluctuations or neural response differences. Other participants, including K2, K3, K4, K5, K7, and K8, demonstrated consistently high metrics, with F1, recall, and precision all exceeding 93%, underscoring the robustness and generalizability of the proposed hybrid EEG+NIRS classification system. These findings confirm the system’s efficacy across diverse individuals and reinforce its potential for reliable multi-class brain-computer interface applications.

### Classification performance using alternative feature extraction and classification methods

We also extracted FWHT, BP, SF-based features from 2.4 s time segments for each modality and classified the extracted features using k-NN to show the effectiveness of using HT features for the hybrid modality. The average test CA results for 8 subjects calculated for each modality are shown in Table 2. In this table, the SD values are given as ± next to the CAs. More effective results are obtained with HT-based features compared to FWHT, BP and SF, as shown in Table 2. The results have shown that HT is effective for signal discrimination in the experimental paradigm proposed in this study.

**Table 2:** Average CA for HT, FWHT, BP, and SF-based features extracted from 2.4-second time segments using the k-NN classifier.

Modalities	Feature Extraction Methods			
	HT	FWHT	BP	SF
**EEG+Deoxy**	96.23 ± 1.30	54.30 ± 2.20	95.31 ± 1.40	51.26 ± 2.55
**EEG + Oxy**	96.28 ± 1.30	54.19 ± 2.26	95.21 ± 1.29	51.22 ± 2.68
**EEG**	91.25 ± 2.64	52.20 ± 2.51	90.05 ± 2.54	47.29 ± 2.74
**Oxy**	55.21 ± 2.70	26.32 ± 2.09	48.81 ± 3.05	25.62 ± 2.05
**Deoxy**	77.31 ± 2.53	29.16 ± 2.23	58.71 ± 2.74	24.94 ± 2.21

HT was employed in this study to accurately capture the distinct spectral and temporal features of EEG and NIRS signals. For EEG, HT was used after a band-pass filter isolated the alpha, beta, theta, and delta frequency bands, enabling analysis of frequency-specific neural oscillations. This approach facilitated the extraction of instantaneous amplitude and phase information, providing a detailed characterization of neural dynamics. In the case of NIRS signals, a low-pass filter was applied before HT to remove high-frequency noise, allowing for the analysis of the hemodynamic fluctuations associated with oxygenated and deoxygenated hemoglobin levels. By computing the instantaneous amplitude and phase, HT revealed key insights into the temporal dynamics of cerebral oxygenation and blood flow. The optimized use of HT, combined with appropriate filtering, allowed for precise extraction of frequency-specific and physiologically relevant information from both EEG and NIRS data, enhancing the sensitivity of the analysis and offering a deeper understanding of the underlying neural and hemodynamic processes.

The superior performance of the HT compared to FWHT, BP, and SF methods in this study can be attributed to its unique ability to accurately capture both spectral and temporal characteristics inherent in the hybrid EEG+NIRS signals. Unlike FWHT and BP, which may primarily emphasize frequency-domain information without fully preserving instantaneous temporal dynamics, HT facilitates the extraction of instantaneous amplitude and phase, providing a detailed representation of transient neural and hemodynamic fluctuations. Thus, the HT’s capacity to reflect fine-grained, physiologically relevant signal features render it especially effective in discriminating between cognitive states within the proposed scrolling text reading paradigm.

The ANOVA test was performed to assess the differences in CA between the different modalities for EEG, EEG + Oxy, EEG+Deoxy, Oxy and Deoxy. The results showed a significant difference in CA between the modalities (p < 0.05). Further analysis showed that EEG+Deoxy and EEG + Oxy had significantly higher classification accuracies than EEG, Oxy and Deoxy (p < 0.05). These results highlighted the effectiveness of combining EEG with NIRS modalities, particularly EEG + Oxy and EEG+Deoxy, in improving classification performance. The significant differences between these hybrid modalities and the standalone modalities supported their potential to improve classification outcomes.

In contrast, the FWHT, BP and SF methods showed significantly poorer performance, especially for the SF-based features, which had the worst accuracies of all modalities. The FWHT and BP methods, although slightly better than SF, still underperformed compared to HT. These suboptimal results can be attributed to the inherent characteristics of the SF-based features and the specific challenges posed by the FWHT and BP methods in capturing the complex, multimodal relationships within the EEG+NIRS data. In particular, SF features may not fully capture the temporal or spatial dynamics of the signals, resulting in lower CA. In addition, the BP and FWHT methods may not effectively exploit the rich temporal dependencies in the data, which are better captured by the HT dependencies method. The classification of HT-based features extracted from 2.4-second time segments for each subject was performed using SVM, DTA, and RF classifiers. The average CA results for eight subjects, obtained from 50 independent runs, are summarized in Table 3. These findings reveal that SVM, DTA, and RF classifiers yielded lower performance compared to the k-NN classifier, emphasizing the effectiveness of k-NN for the hybrid EEG+NIRS dataset.

**Table 3:** Average ca for HT-based features extracted from 2.4-second time segments using classification methods.

Modalities	Classification Methods		
	SVM	DTA	RFA
**EEG+Deoxy**	95.40 ± 1.52	56.75 ± 4.77	74.98 ± 3.90
**EEG + Oxy**	95.51 ± 1.47	65.04 ± 6.43	75.81 ± 5.12
**EEG**	91.46 ± 1.93	71.52 ± 2.90	87.71 ± 3.15
**Oxy**	58.27 ± 2.46	43.76 ± 3.01	53.30 ± 2.84
**Deoxy**	79.14 ± 2.30	57.93 ± 3.20	75.94 ± 2.92

The classification of HT-based features extracted from 2.4-second time segments for each subject was performed using SVM, DTA, and RF classifiers. The average CA results for eight subjects, obtained from 50 independent runs, are summarized in Table 3. These findings reveal that SVM, DTA, and RF classifiers yielded lower performance compared to the k-NN classifier, emphasizing the effectiveness of k-NN for the hybrid EEG+NIRS dataset.

Additionally, considering the recent prominence of LSTM networks in signal classification, this approach was applied to the dataset to evaluate its suitability [32,33]. However, the classification accuracies across all modalities were notably low, ranging between 20% and 25%. This suboptimal performance may be attributed to several factors intrinsic to the EEG+NIRS data. Specifically, the complex multimodal signals exhibit temporal and structural characteristics that may not align well with the sequential learning mechanisms of standard LSTM architectures. It is plausible that the current LSTM configuration lacks the necessary depth or architectural adaptations to effectively capture the nuanced temporal dependencies and cross-modal interactions present in the data. Consequently, these findings underscore the importance of tailoring deep learning architectures to the unique properties of hybrid neurophysiological datasets. Future work will explore alternative architectures, including convolutional recurrent networks, transformer-based models, or customized hybrid frameworks, which may better exploit the rich spatiotemporal features inherent in EEG and NIRS signals to improve classification outcomes.

### Computational time analysis of time

We calculated the computational time of feature extraction for a single trial of all time segments for each modality, and it is presented in Table 4 in milliseconds (msec). When analyzing these findings, it is found that 2.4-sec time segments produce the best results with the hybrid modality. As can be seen, as the time segment increases, the computational time of feature extraction for a single trial also increases. Also, it should be noted that, as seen in Figs 8, 9, 10, and 11, as the time segment increases in terms of seconds, it can be inferred that the test CA decreases. Processing shorter signals in terms of time reduces computational complexity and requires less storage space. When all of this is considered, it is clear that using 2.4-sec signal segments is more appropriate for BCI systems based on reading scrolling text in the right, left, up, and down directions.

**Table 4:** The computational time of feature extraction for a single trial across different time segments.

Modalities	Time Segments			
	2.4 sec	4.8 sec	12 sec	24 sec
**EEG+Deoxy**	0.810	0.842	1.013	1.151
**EEG + Oxy**	0.813	0.844	1.006	1.035
**EEG**	0.781	0.823	0.691	0.889
**Oxy**	0.020	0.031	0.053	0.077
**Deoxy**	0.020	0.031	0.035	0.078

### Comparison of the results with existing literature

The proposed versatile scrolling text reading paradigm-based hybrid EEG+NIRS BCI system was comprehensively compared with existing studies in the literature, considering experimental paradigms, EEG and NIRS features, classifier control commands and CA. As shown in Table 5, the proposed method achieved a CA of 96.28% using a hybrid EEG+NIRS framework and a k-NN classifier, significantly outperforming most studies. Notably, the number of control commands played a critical role in determining the performance of different studies. For example, studies such as [34,36] and [37] reported CAs ranging from 61.30% to 88.10%, despite using different experimental paradigms and classification techniques. In particular, the study in [18], which also used a multi-command paradigm, achieved a CA of 79.31%, further highlighting the advantage of the Hilbert transform-based

**Table 5:** Studies in the literature are compared to the proposed method.

References	Procedure					CA(%)	
	Experiment Paradigm	NIRS Features	EEG Features	Classifier	Control Command	EEG	Hybrid
[34]	MI: Imagination of right/left-hand movement	Average Amplitude	CSP and Logarithmic Power	LDA	2	57.7	61.30
[35]	MI: Imagination of right/left-hand movement	Mean and slope	CSP	LDA	2	85.20	94.20
[36]	MI: Imagination of right/left-hand movement	Hurst Exponent	CSP	SVM	2	74.7	81.20
[16]	ME: Right/left-hand movement	Mean	Wavelet Transform	SVM	2	85.64	91.02
[37]	MA: Mental processing/ rest state	Slope and Variance	CSP	LDA	3	75.90	88.10
[18]	MA: Memorizing four, five and six-digit numbers and remembering them in the task	Mean	Band Power	LDA	4	65.52	79.31
[26]	MI: Imagination of right/left-hand movement	FWHD	FWHD	*k*-NN	2	71.46	78.21
[38]	MI: Imagination of right/left-hand movement	Multiple Bandwidth Method	Multiple Bandwidth Method	Convolutional Neural Networks	2	98.72	99.85
Proposed Method	Reading left, right, up and down scrolling text	Hilbert transform	Hilbert transform	*k*-NN	4	91.25	96.28

feature extraction employed in the proposed method. This technique effectively captures the complex dynamics of EEG and NIRS signals during multitask cognitive operations.

In comparison, while the study in [35] achieved a CA of 94.20%, it was limited to a binary MI task, which is considerably less complex than the four-command paradigm used in this research. Furthermore, the study in [38] reported an impressive CA of 99.85%, but it focused on a simpler two-class problem, which does not pose the same level of challenge as multi-command tasks. The proposed method, with a CA 16.97% higher than the best performing four-class study, underlines its robustness and effectiveness in tackling more complex tasks. The results also showed significant improvements when combining EEG with NIRS features in hybrid systems, as opposed to relying on EEG-based methods alone. Studies such as [34,36] and [37] showed modest improvements in CA with hybrid systems, with [34] improving from 57.7% to 61.30%, [36] from 74.7% to 81.20% and [37] from 75.90% to 88.10%. The hybrid system in [35] achieved 94.20%, a significant increase from 85.20% with EEG alone. Similarly, the hybrid approaches in [16] and [18] achieved CAs of 91.02% and 79.31%, respectively, compared to 85.64% and 65.52% for EEG alone. In contrast, the proposed method using the four-command scrolling text reading paradigm and Hilbert transform for feature extraction resulted in a CA of 96.28%, surpassing all previous results. These results not only highlight the superior performance of the hybrid EEG+NIRS system in multi-command tasks, but also emphasize its computational efficiency, making it suitable for resource-constrained environments. The high classification accuracy achieved in this study demonstrates the potential of this method to advance real-world BCI applications. In addition, the creation of a novel multi-class EEG+NIRS dataset for this study contributes valuable resources to the literature and provides a strong foundation for future BCI innovations.

## Conclusion

In this study, using a hybrid modality of the original dataset, we proposed a high-performance BCI system based on reading scrolling text in different directions (right, left, up and down). In addition, with a special focus on widely used signal processing and machine learning algorithms, the potential impact of the experimental procedure based on the scrolling text reading paradigm on the hybrid BCI system was investigated. Classification of HT-based features extracted from 2.4-sec time segments of the multimodal signals recorded during scrolling text reading using k-NN was proposed for this study. The results showed that the proposed method provided a mean test CA of %96.28 ± 1.30 for the EEG + Oxy modality. The method demonstrated superior classification performance in most cases when compared with different approaches and hybrid studies in the literature. Furthermore, through the experimental paradigm based on reading scrolling text, it is believed that the obtained results will shed light on the development of a hybrid EEG+NIRS-based BCI system. The empirical results confirm the commendable performance of this framework. This represents a notable step forward in the field of BCI. Another noteworthy aspect of this study was the data augmentation through time segmentation. This not only allowed for more reliable data analysis during the train phase, but also provided faster command outputs to the BCI system.

Moreover, the demonstrated high classification performance based on short time windows enables faster and more efficient command generation, which is critical for real-time BCI applications. This capability is particularly promising for individuals with severe motor impairments, such as ALS patients, as it supports the development of intuitive and responsive communication interfaces. The findings thus bridge the gap between theoretical research and practical utility, reinforcing the potential of the proposed system for real-world assistive BCI technologies.

While the current study primarily focuses on offline analysis to establish the efficacy of the proposed hybrid BCI system, the promising results lay a strong foundation for future investigations into real-time implementation. Transitioning from offline to online environments involves addressing challenges such as minimizing latency, ensuring prompt and accurate signal processing, and maintaining system robustness under dynamic conditions. The high classification accuracy achieved within short temporal windows in this study suggests that the proposed approach has inherent potential to support rapid command generation, which is critical for responsive and user-friendly BCI applications.

It is acknowledged that the relatively small sample size of eight human participants may limit the generalizability of the current findings. Nevertheless, this exploratory study provides a valuable proof-of-concept demonstrating the effectiveness of the proposed hybrid BCI framework in human subjects. Future research will aim to recruit a larger and more diverse group of participants to validate and extend these results, thereby enhancing the robustness and applicability of the system across broader populations.

## Discussion

This study used a scrolling text reading paradigm to investigate the effects of different scrolling directions on neural activity. It builds on the theoretical framework that suggests that cognitive tasks such as visual motion perception, language processing and attentional control are governed by specialized yet interconnected neural mechanisms. The interconnectivity of these mechanisms highlights the brain’s remarkable ability to coordinate multiple regions, facilitating the execution of complex cognitive functions with precision and efficiency. In a related previous study [39], we investigated neural activation using a hybrid dataset combining EEG + Oxy+Deoxy signals. This approach yielded a CA of 95.54%, highlighting the significant potential of the hybrid EEG + Oxy+Deoxy dataset for advanced neural activity analysis.

Future research will aim to comprehensively evaluate the hybrid EEG + Oxy model proposed in this study under different experimental conditions. In particular, a more detailed investigation of how different cognitive tasks and scrolling directions influence neural activity is expected to contribute to both theoretical understanding of neural processes and practical applications. The effective use of hybrid datasets represents a promising avenue for advancing sophisticated analytical methods and deepening insights into neural mechanisms.

Furthermore, the study highlights the significant potential of hybrid EEG+NIRS systems in BCI applications. With a CA of 96.28%, the proposed system outperforms most hybrid-mode systems reported in the literature, demonstrating the benefits of integrating complementary modalities. By exploiting the strengths of both EEG and NIRS, the system demonstrates robust neural decoding capabilities for multi-command tasks, advancing the applicability of hybrid BCIs.

A key contribution of this research is the systematic analysis of data length optimization for feature extraction and classification. The results identified a 2.4 second window as optimal, achieving the highest CA by balancing temporal resolution and noise suppression. Shorter data lengths were found to miss critical temporal patterns essential for accurate classification, while longer durations introduced variability and redundancy, compromising the discriminative power of the features. These findings highlight the importance of selecting an appropriate data length to maximize system performance and maintain computational efficiency.

In conclusion, this study presents a novel hybrid EEG+NIRS-based BCI system using a scrolling text reading paradigm that achieves state-of-the-art classification performance. The results highlight the feasibility and effectiveness of hybrid systems for multi-command tasks, with significant implications for the advancement of neurotechnological applications. By addressing limitations and paving the way for future investigations, this research lays a solid foundation for the development of more robust and versatile BCI systems, fostering advances in neuroscience and assistive technologies.
